# Psychosomatic aspects of psoriasis and atopic dermatitis

**DOI:** 10.1192/bjo.2021.80

**Published:** 2021-06-18

**Authors:** Olga Belugina

**Affiliations:** Belarusian State Medical University

## Abstract

**Aims:**

The aim of this study is to assess the level of alexithymia, coping strategies and stress contribution to illness in patients with psoriasis and atopic dermatitis in order to increase effectiveness of dermatological treatment.

**Method:**

59 patients with atopic dermatitis, 67 with psoriasis and 65 healthy control group individuals were included in the cross-sectional study. Predominant complains of the patients: itching, widespread rashes and rashes on the open areas of the skin. In 85% patients with skin pathology onset of the disease and relapses were associated with stress, in 15% other factors.

“The 20-item Toronto Alexithymia Scale” was used to assess alexithymia. “The Ways of Coping Checklist, Lazarus” was used to assess coping-strategies. “The Holmes and Rage Stress Inventory” was used to assess stress contribution to illness. Significance level: p < 0,05.

**Result:**

The levels of alexithymia (p = 0.002), difficulty identifying feelings subscale (p = 0.02) and externally-oriented thinking subscale (p = 0.002) in patients with skin pathology (especially in those with psoriasis) were higher than in the control group.

Patients with skin pathology turned out to be more susceptible to stress factors (p = 0.025) and less often use coping strategy “seeking social support” (p = 0.037).

Patients with skin pathology with high levels of alexithymia and difficulty identifying feelings subscale more likely to use maladaptive “escape-avoidance” coping (p = 0.001).

Patients with atopic dermatitis who find difficult to describe feelings are more likely to use maladaptive coping “distancing”(p = 0.002).

In patients with psoriasis high levels of alexithymia and externally-oriented thinking subscale scores are associated with less common use of the adaptive coping “problem solving”(p = 0.001). Moreover, in patients with psoriasis high levels of difficulty identifying feelings subscale are associated with more common use of maladaptive “escape-avoidance” coping (p = 0.001).

**Conclusion:**

The results of the study confirm the need to include psychological assessment and psychotherapy in the treatment plan for patients with psoriasis and atopic dermatitis in order to improve emotional awareness and to develop more adaptive coping-strategies in patients.

